# Healing of rectal advancement flaps for anal fistulas in patients with and without Crohn’s disease: a retrospective cohort analysis

**DOI:** 10.1186/s12893-021-01282-4

**Published:** 2021-06-05

**Authors:** Claudia Seifarth, Kai S. Lehmann, Christoph Holmer, Ioannis Pozios

**Affiliations:** 1 Charité–Universitätsmedizin Berlin, corporate member of Freie Universität Berlin, Humboldt-Universität Zu Berlin and Berlin Institute of Health, Department of General-, Visceral- and Vascular Surgery, Berlin, Germany; 2 Department of General and Visceral Surgery, St. Joseph Krankenhaus, Berlin, Germany

**Keywords:** Anal fistula, Rectal advancement flap, Crohn’s disease

## Abstract

**Background:**

Surgical closure of anal fistulas with rectal advancement flaps is an established standard method, but it has a high degree of healing failure in some cases. The aim of this study was to identify risk factors for anal fistula healing failure after advancement flap placement between patients with cryptoglandular fistulas and patients with Crohn’s disease (CD).

**Methods:**

From January 2010 to October 2020, 155 rectal advancement flaps (CD patients = 55, non-CD patients = 100) were performed. Patients were entered into a prospective database, and healing rates were retrospectively analysed.

**Results:**

The median follow-up period was 189 days (95% CI: 109–269). The overall complication rate was 5.8%. The total healing rate for all rectal advancement flaps was 56%. CD patients were younger (33 vs. 43 years, p < 0.001), more often female (76% vs. 30%, p < 0.001), were administered more immunosuppressant medication (65% vs. 5%, p < 0.001), and had more rectovaginal fistulas (29% vs. 8%, p = 0.001) and more protective stomas (49% vs. 2%, p < 0.001) than patients without CD. However, no difference in healing rate was noted between patients with or without CD (47% vs. 60%, p = 0.088).

**Conclusions:**

Patients with anal fistulas with and without Crohn’s disease exhibit the same healing rate. Although patients with CD display different patient-specific characteristics, no independent factors for the occurrence of anal fistula healing failure could be determined.

*Trial registration* Not applicable due to the retrospective study design.

## Background

Treatment of anal fistulas is difficult and is associated with high rates of healing failure, sphincter damage, incontinence, and impaired quality of life. A variety of treatment options exist, including fistulotomy for superficial fistula course or seton drainage [1, 2]. Fistula closure can also be performed using surgical reconstructive and sphincter-preserving methods, such as mucosal or submucosal rectal advancement flap or LIFT (ligation of intersphinteric fistula tract) procedures [3–6]. The results after LIFT or advancement flap were examined separately in a large review for cryptoglandular and CD fistulas with comparable results [6]. However, no comparison between cryptoglandular and CD fistulas was performed in this study. In addition, biomaterials, such as fibrin, collagen, or even autologous stem cells, have also been developed for fistula closure [7, 8]. Despite the large number of different treatment options, no procedure has achieved a breakthrough in the treatment of anal fistulas, and the healing rates remain unsatisfactory. Wide ranges of healing rates for the above procedures have been reported in the literature. The rectal advancement flap exhibits the best healing rates (between 30 and 100%) [9–11]. Previous studies described poor results after flap advancement in patients with active Crohn's disease [12, 13]. Proctitis or stenosis should therefore be resolved before advancement flap procedures are performed. If inflammation is present, systemic or topical therapy should be administered, especially in CD patients.

To date, studies regarding healing rates of anal fistulas in CD patients are rare [14, 15]. Prospective studies date from the 1990s and examine only a small number of CD patients. In addition, only a few studies, some of which have small case numbers, compare the results of an advancement flap for CD-associated anal fistulas and cryptoglandular anal fistulas [13, 16, 17]. A limited number of retrospective studies on CD anal fistulas address various surgical treatments, including seton drainage and fistulotomy, without a focus on advancement flaps [18].

The primary aim of this study was to compare the healing rate after the use of rectal advancement flaps for anal fistulas in patients with cryptoglandular fistulas and patients with Crohn’s disease. The second aim was to identify risk factors for healing failure in CD patients.

## Methods

### Patients

The study protocol was approved by the Medical Ethical Committee of Charité–Universitätsmedizin Berlin (EA4/149/20). From January 2010 to October 2020, 155 rectal advancement flaps were performed consecutively for patients with anal fistula in Charité–Universitätsmedizin Berlin, Campus Benjamin Franklin. Data analysis included both CD patients and patients with cryptoglandular fistulas. The inclusion criteria were all patients with rectal advancement flaps for anal fistula. Exclusion criteria were age under 18 years and surgical treatments (seton drainage, fistula cutting, stem cell therapy, and gracilis flap) other than rectal advancement flap. In CD patients, the indication for an advancement flap was a healed perianal abscess and a CD-free rectal mucosa. All rectal advancement flaps were performed by surgeons experienced in proctology and CD in our department. In this technique, the internal fistula opening is closed with a flap including the lamina muscularis and the mucosa of the rectum wall. The external opening of the fistula is excised or debrided. No additional surgery was performed during fistula repair. As planned, our patients came for their first follow-up after four to six weeks. CD patients were consulted more often. No scheduled follow-up was conducted after the healing was complete. Patients were only examined more frequently in cases of failure to heal.

### Aim, design, and settings

Data were collected from the hospital's electronic patient record system (EHS) in a prospective database and retrospectively evaluated. The primary aim was fistula healing. It was defined as complete healing of the fistulous tract (clinically and in anal endoscopic ultrasound) without the need for reoperation or replacement of the seton drain. In contrast, healing failure was defined as evidence of a recurrent fistula that required at least seton drainage or reoperation. Healing failure included persisting fistula (early recurrence) and recurrence of new fistula (late recurrence). Lost to follow-up was defined as any lack of contact after patient discharge. For evaluation, patient age, diagnosis, sex, immunosuppressant medication, ASA (American Society of Anaesthesiologists) score, body mass index (BMI), rectovaginal fistula course, protective stoma, and complication rate (Clavien-Dindo classification) were documented. Nicotine and alcohol abuse were not recorded due to the lack of documentation.

### Statistics

Given that most variables did not show a normal distribution, nonparametric tests were used for statistical comparison. Continuous variables are displayed as medians (minimum–maximum), and categorical variables are displayed as counts (percentages). The Mann–Whitney U test was used to compare two independent groups. Group comparisons for categorical variables were performed using the chi-square test. The level of significance was 0.05 (2-sided) for each statistical test. P-values concerning secondary endpoints were considered exploratory and are presented without Bonferroni correction. Factors with P-values less than 0.2 were enrolled in a Cox hazard regression model to identify independent risk factors. Kaplan–Meier estimates were calculated for the healing rate with the last available contact date (follow-up = time to event). The log rank test was used for the comparison between patients without and with CD. We assumed that loss to follow-up was missing not at random (MNAR) and did not address this with specific statistical measures. Statistical analysis was performed using SPSS Statistics Software 25.0 (IBM, Armonk, NY, USA).

## Results

### Patients

Between January 2010 and June 2020, 155 mucosal advancement flap operations for patients with anal fistula were performed (Table 1). The study included 55 CD patients and 100 non-CD patients. Most patients had complex fistulas. In total, 83% had a transsphincteric fistula, and 15% had a rectovaginal fistula course. Immunosuppressant medication was administered to 41 patients (26%). Median healing over all flaps was 56%. Nine patients (5.8%) developed acute complications (haematoma, bleeding) with the need for reperform surgery. Two patients (1%) were lost to follow-up.

**Table 1:** General patient data over all advancement flaps

	n = 155
Pathogenesis	
Crohn’s disease	55 (35)
Cryptoglandular	100 (65)
Fistula course	
Rectovaginal	24 (15)
Transsphincteric	128 (83)
Suprasphincteric	2 (1)
Intersphincteric	1 (1)
Ostomy	39 (25)
Age, years	40 (12–73)
Sex, female	71 (46)
ASA, 1–2	144 (93)
BMI, m^2^/kg	25 (17–44)
Immunosuppressant medication	41 (26)
Anti-TNF	28 (18)
Anti-Interleukin	1 (0.5)
Anti-Integrin	5 (3)
Other	7 (4.5)
Fistula healing	86 (56)
Complications	9 (5.8)
Mortality	0
Follow-up, days (95% CI)	189 (109–269)

Median (min–max) for continuous variables, count (percentage) for categorical variables, except for Follow-up estimates by Kaplan Meier: median (95% CI)

*ASA Score* = American Society of Anesthesiologists Score, *BMI*  = Body mass index

### Risk of anal fistula healing failure after advancement flap for all patients

In Table 2, univariate analysis showed that female sex, immunosuppressant medication, and rectovaginal fistula course were significant influencing factors for healing failure. Crohn’s disease, BMI, ASA 1 and 2, or the presence of protective stoma showed no influence on anal fistula healing failure. P-values less than 0.2 from the univariate analysis were enrolled in a Cox proportional hazard model to identify independent risk factors for healing failure. Cox regression analysis could not identify any independent influencing factor on healing after rectal advancement flap placement.

**Table 2:** Factors affecting anal fistula healing failure in all patients

	Healing n = 86 (56%)	Healing failure n = 69 (44%)	Missing	P-value	Cox regression	
					Hazard ratio (95% CI)	P-value
Age, years	43 (12–73)	36 (14–64)	0	0.063	0.992 (0.973–1.010)	0.377
Sex, female	32 (37)	39 (56)	0	0.013	1.515 (0.847–2.707)	0.161
BMI, m^2^/kg	25 (17–42)	25 (17–44)	4	0.460	–	–
ASA, 1–2	78 (91)	66 (97)	0	0.191	0.779 (0.233–2.600)	0.685
Immunosuppression, yes	17 (20)	24 (35)	0	0.027	0.894 (0.422–1.896)	0.771
Pathogenesis Crohn’s disease Other	26 (30) 60 (70)	29 (42) 40 (58)	0	0.088	0.884 (0.423–1.849)	0.743
Rectovaginal fistulas	9 (38)	15 (62)	0	0.044	0.675 (0.341–1.336)	0.259
Protective stoma	19 (22)	20 (29)	0	0.213	–	–

*ASA* *Score* = American Society of Anesthesiologists Score, *BMI *= Body mass index

### Advancement flap in CD patients

Table 3 shows the modified Montreal Classification [19] for all CD patients. Differences in the characteristics of patients with and without CD are presented in Table 4. CD patients were significantly younger, were more often female, received more immunosuppressant medication, and had a lower BMI than non-CD patients. In addition, CD patients were more likely to have a protective ostomy. There were significantly more patients with rectovaginal fistulas among CD patients. However, the healing rate of anal fistula did not differ between CD and non-CD patients (p = 0.088).

**Table 3:** Modified montreal classification [19]

	n = 55
Age at diagnosis	
A1 (< 16 years)	2 (3.6)
A2 (17–39 years)	34 (61.8)
A3 (> 40 years)	19 (34.6)
Location of disease	
L1 (ileal)	0
L2 (colon)	0
L3 (ileocolonic)	0
L4 (upper disease)	0
Anal/perianal	55 (100)
Behavior	
B1 (inflammatory)	0
B2 (stricturing)	0
B3 (penetrating)	0
Fistulas	55 (100)

**Table 4:** Characteristics for patients without and with Crohn’s disease (CD)

	CD patients n = 55	Non-CD patients n = 100	Missing	P-value
Age, years	33 (14–66)	43 (12–73)	0	< 0.001
Sex, female	41 (75)	30 (30)	0	< 0.001
BMI, m^2^/kg	24.4 (17.3–34.9)	25.6 (16.5–43.6)	4	0.033
ASA, 1–2	54 (98)	90 (90)	0	0.051
Immunosuppression, yes	36 (65)	5 (5)	0	< 0.001
Rectovaginal fistula	16 (29)	8 (8)	0	0.001
Ostomy	27 (49)	12 (12)	0	< 0.001
Fistula healing	26 (47)	60 (60)	0	0.088
Follow-up, days (95% CI)	210 (53–368)	89 (111–267)	0	0.360

*CD* = Crohn’s disease, *ASA Score* = American Society of Anesthesiologists Score, *BMI* = Body mass index. Follow-up estimates by Kaplan Meier: median (95% CI)

A subgroup analysis was performed for CD patients to identify possible influencing factors for anal fistula healing failure (Table 5). Neither age nor sex, sex, BMI, ASA, or immunosuppressant medication showed a significant influence on fistula healing after flap advancement. A rectovaginal fistula course or the presence of a protective stoma was also irrelevant to the healing process. P-values less than 0.2 from the univariate analysis were enrolled in a Cox proportional hazard model to identify independent risk factors for healing failure. Cox regression analysis could not find any independent influencing factor on healing after rectal advancement flap placement.

**Table 5:** Factors for healing failure of advancement flaps in CD patients

	Healing n = 26	Healing failure n = 29	P-value	Cox regression	
				Hazard ratio (95% CI)	P-value
Age, years	33 (20–66)	31 (14–54)	0.428	–	
Sex, female	17 (65)	24 (82)	0.122	2.537 (0.881–7.303)	0.085
BMI, m^2^/kg	24.5 (17.3–33.7)	24.4 (19.1–34.9)	0.873	–	
ASA, 1–2	25 (96)	29 (100)	0.473	–	
Immunosuppressant medication	14 (54)	22 (76)	0.076	1.096 (0.447–2.688)	0.841
Rectovaginal fistula	5 (19)	11 (38)	0.109	0.876 (0.366–2.097)	0.766
Ostomy	12 (46)	15 (52)	0.444	–	

*ASA Score* = American Society of Anesthesiologists Score, *BMI* = Body mass index

### Healing failure

**Fig. 1:**
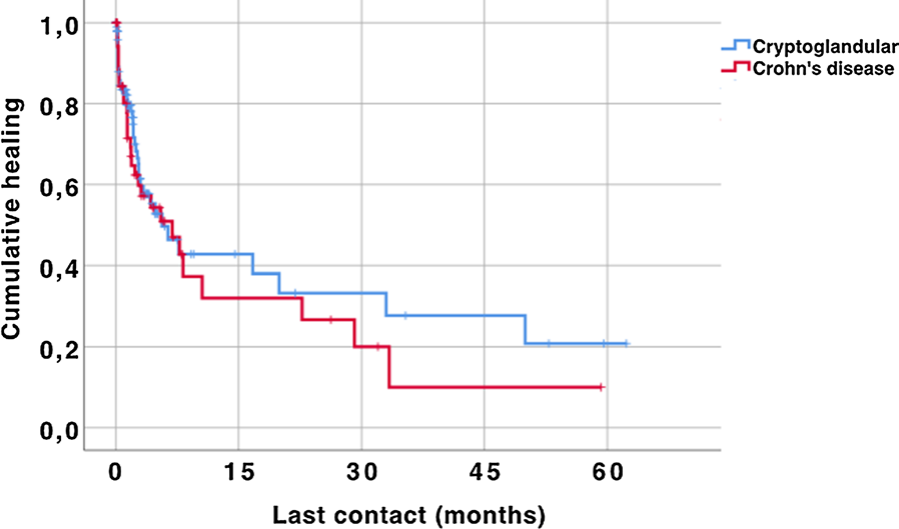
Kaplan–Meier estimates for fistula healing. P-value: 0.500

The median follow-up for CD patients was 210 days (95% CI: 53–368). Non-CD patients had a median follow-up of 89 days (111–267). Two patients (1%) were lost to follow-up. Healing failure occurred in 69 (44%) of 155 advancement flaps. Kaplan–Meier estimates for fistula healing failure did not differ between patients with cryptoglandular fistulas and patients with CD (Fig. 1).

The CD and non-CD patients with anal fistula healing failure were further classified into two categories according to the time of relapse. An early relapse was reported within 14 days and is equivalent to a persisting fistula. A late relapse was reported after 14 days. Of 29 CD patients with anal fistula healing failure, 8 patients (28%) had a median early relapse of 10 days (6–12), and 21 patients (72%) had a median late relapse of 84 days (29–1016). Of 40 non-CD patients with anal fistula healing failure, 14 (35%) patients had a median early relapse of 8 days (4–14), and 26 patients (65%) had a median late relapse of 85 days (16–1521). The difference for both early (p = 0.552) and late (p = 0.082) healing failure was not significant.

## Discussion

Patients with Crohn’s disease (CD) are a special and demanding group of patients with a known increase in perioperative morbidity [20, 21]. This also includes the surgical treatment of CD-associated anal fistulas. Given limited evidence on healing rates after advancement flaps in CD [15, 22–24], we aimed to analyse potential risk factors for healing failure in this particular group of patients.

We showed that CD patients significantly differed from patients with cryptoglandular fistulas, especially concerning increased immunosuppression and the presence of ostomy. Nevertheless, CD patients did not have an increased risk of healing failure compared with patients with cryptoglandular fistulas. Although CD patients were significantly younger and were more often female, had a smaller BMI and were taking immunosuppressant drugs more frequently, no independent risk factors for healing failure of anal fistula after advancement flap were identified. This is a comparative study on this subject. In the past, most studies dealt with either only CD patients or only cryptoglandular fistulas [4, 22, 25].

Various risk factors for failure of fistula healing have been reported in the literature. Previous work identified obesity as a risk factor for healing failure of anal fistulas [26, 27]. This statement is not consistent with our results. Our results showed that non-CD patients had a significantly higher BMI than CD patients. However, no influence of BMI on anal fistula healing was noted in either CD or non-CD patients. Therefore, it remains unclear whether BMI affects healing after rectal advancement flaps.

Several technical options are available for rectal advancement flaps. Simple mucosal, partial or full-thickness flaps are described in the literature, and analyses concerning their possible healing failure have been reported [28]. The best results are described for full-thickness flaps [30]. The technique used in our patients was most similar to the partial thickness flap with moderate results. We prefer this technique to avoid major tissue defects and injuries to the sphincter.

Another common risk factor for postoperative healing disorders is nicotine abuse, especially in CD patients [29, 30]. Previous data showed that there was no influence of nicotine, even excessive smoking, on anal fistula recurrence [31, 32]. We did not assess this variable in our work. As this is a retrospective data analysis, this value was not fully documented and could therefore not be adequately assessed.

Rectovaginal fistulas are associated with healing disorders after anal fistula repair. Different studies have demonstrated this effect for different surgical therapies, such as internal and external flaps [15, 33]. In the case of persistent, complicating, or recurrent abscessing fistulation, proctectomy is necessary for some patients [34]. Indeed, rectovaginal fistulas occurred more frequently in CD patients in our study. However, our analyses showed that this factor did not affect the healing rate. Therefore, we believe that the inclusion of patients with rectovaginal fistulas is feasible and does not introduce a significant bias. Over the years, particularly difficult and complex rectovaginal fistulas have been treated more often with other surgical techniques, such as gracilis flaps, in our department. This phenomenon could explain the lower healing rates of rectovaginal fistulas after rectal advancement flaps in our study. It should also be mentioned that in addition to the increasing indication for primary gracilis flap in difficult and complex rectovaginal fistulas, at least in the case of a recurrence of a rectovaginal fistula, the gracilis flap should be used for fistula closure [35]. Furthermore, the application of transanal minimally invasive surgery (TAMIS) for advancement rectal flaps could improve the healing outcome even in difficult situations (such as high rectal fistulas) [36]. Treatment of anal fistula with gracilis flap or Martius flap was not assessed in this study but is planned for further work.

Proximal bowel diversion was previously reported to be associated with lower recurrence rates after anal fistula surgery. However, studies do not agree on whether and when a positive effect can be achieved depending on the severity of the underlying disease [37, 38]. In our work, CD patients were more likely to have a protective stoma, but this did not affect the healing rate. It can therefore be assumed that other factors, such as the course of the fistula or the severity of Crohn's disease, may play a role in fistula healing.

One limitation of the study is its retrospective nature. In addition, the heterogeneous group of patients, including CD and non-CD patients as well as patients with complex fistulas, seems to represent a further limitation. On one hand, we divided CD and non-CD patients and analysed these patients separately. On the other hand, complex fistulas are typical for CD patients and should therefore be specifically included in the analysis. Including all the patients above, it was possible to conduct a detailed analysis for rectal advancement flaps with a high number of patients achieving important results generally for anal fistula treatment, particularly CD patients. Furthermore, we did not perform a power analysis a priori, so we can only surmise whether the analysis is statistically powered. To the best of our knowledge, this study includes the greatest number of patients in total and CD patients in particular on this subject.

## Conclusions

Patients with Crohn’s disease often present with complicated anal fistulas, increased immunosuppression, and ostomy. However, the healing rate after rectal advancement flap placement is similar to that of patients with cryptoglandular fistulas.

## Data Availability

The datasets used and/or analyzed during the current study are available from the corresponding author on reasonable request.

## Abbreviations

CD: Crohn’s disease
CI: Confidence interval
LIFT: Ligation of intersphinteric fistula tract
ASA: American Society of Anesthesiologists score
BMI: Body mass index
MNAR: Missing not at random
